# Diagnostic performance and characteristics of anterior nasal collection for the SARS-CoV-2 antigen test: a prospective study

**DOI:** 10.1038/s41598-021-90026-8

**Published:** 2021-05-18

**Authors:** Yuto Takeuchi, Yusaku Akashi, Daisuke Kato, Miwa Kuwahara, Shino Muramatsu, Atsuo Ueda, Shigeyuki Notake, Koji Nakamura, Hiroichi Ishikawa, Hiromichi Suzuki

**Affiliations:** 1 Department of Infectious Diseases, University of Tsukuba Hospital, 2-1-1 Amakubo, Tsukuba, Ibaraki 3058576 Japan; 2 Division of Infectious Diseases, Department of Medicine, Tsukuba Medical Center Hospital, 1-3-1 Amakubo, Tsukuba, Ibaraki 3058558 Japan; 3 Akashi Internal Medicine Clinic, 3-1-63 Asahigaoka, Kashiwara, Osaka 5820026 Japan; 4 Gosen Site, Research and Development Division, Reagent R&D Department, Denka Co., Ltd., 1-2-2 Minami-hon-cho, Gosen-shi, Niigata 9591695 Japan; 5 Department of Clinical Laboratory, Tsukuba Medical Center Hospital, 1-3-1 Amakubo, Tsukuba, Ibaraki 3058558 Japan; 6 Department of Respiratory Medicine, Tsukuba Medical Center Hospital, 1-3-1 Amakubo, Tsukuba, Ibaraki 3058558 Japan; 7 Department of Infectious Diseases, Faculty of Medicine, University of Tsukuba, 1-1-1 Tennodai, Tsukuba, Ibaraki 3058575 Japan

**Keywords:** Infectious-disease diagnostics, SARS-CoV-2, Medical research

## Abstract

The clinical utility of antigen test using anterior nasal samples has not been well evaluated. We conducted a prospective study in a drive-through testing site located at a PCR center to evaluate the diagnostic performance of the antigen test QuickNavi-COVID19 Ag using anterior nasal samples and to compare the degrees of coughs or sneezes induction and the severity of pain between anterior nasal collection and nasopharyngeal collection. The study included a total of 862 participants, of which 91.6% were symptomatic. The median duration from symptom onset to sample collection was 2.0 days. Fifty-one participants tested positive for severe acute respiratory syndrome coronavirus 2 on reverse transcription PCR (RT-PCR) with nasopharyngeal samples, and all of them were symptomatic. In comparison to the findings of RT-PCR, the antigen test using anterior nasal samples showed 72.5% sensitivity (95% confidence interval [CI] 58.3–84.1%) and 100% specificity (95% CI 99.3–100%). Anterior nasal collection was associated with a significantly lower degree of coughs or sneezes induction and the severity of pain in comparison to nasopharyngeal collection (*p* < 0.001). The antigen test using anterior nasal samples showed moderate sensitivity in symptomatic patients who were at the early stages of the disease course but was less painful and induced fewer coughs or sneezes.

## Introduction

The laboratory diagnosis of severe acute respiratory syndrome coronavirus 2 (SARS-CoV-2) includes nucleic acid amplification tests (NAATs), antigen tests, and antibody tests^1^. Antigen tests are generally less sensitive than NAATs for detecting SARS-CoV-2 but are relatively inexpensive, and most can be performed at the point of care^2^.

The diagnostic performance of antigen test of nasopharyngeal samples has been evaluated, and the reported specificity is consistently high > 97%, while sensitivity ranges from 0 to 94%^2^. We previously evaluated the performance of an antigen test, QuickNavi-COVID19 Ag (Denka Co., Ltd., Tokyo, Japan), using nasopharyngeal samples from 1186 participants^3^. The overall sensitivity and specificity were 86.7% (95% confident interval [CI] 78.6–92.5%) and 100% (95% CI 99.7–100%), respectively, and the sensitivity for symptomatic participants was 91.7% (95% CI 82.7–96.9%)^3^.

In recent studies, the sensitivity of anterior nasal samples was reported to be comparable to that of nasopharyngeal samples for NAATs^4,5^. Anterior nasal collection was reported to be less painful than nasopharyngeal collection^6^ and may cause less droplet dispersal. These benefits are also expected in antigen tests but have yet to be fully evaluated.

We prospectively evaluated the diagnostic performance of the QuickNavi-COVID19 Ag test using anterior nasal samples and compared the degrees of coughs or sneezes induction and the severity of pain between anterior nasal collection and nasopharyngeal collection.

## Methods

We conducted the present prospective study between October 7, 2020 and January 9, 2021, at a drive-through PCR center where participants were referred from a local public health center and 97 primary care facilities in Tsukuba, Japan. After receiving the participants’ informed consent, additional anterior nasal samples for the antigen test were collected and their clinical information was obtained. Cases with no clinical data were excluded from this study. In cases where participants enrolled in the current study more than once, only the first evaluation was included in this study.

QuickNavi-COVID19 Ag is a lateral-flow antigen test which employs a sandwich immunochromatography method with mouse monoclonal antibodies against SARS-CoV-2. This method has been employed by various other antigen detection tests^2^. After a sample is mixed with specimen buffer and specimen droplets are added to the test cassette well, the monoclonal antibodies on a conjugate pad reacts with SARS-CoV-2 nucleoprotein. The antibody-antigen complexes are then captured by other monoclonal antibodies fixed on the test line, visualizing a red colored test line and that indicates a positive test result.

### Sample collection and the antigen test procedure

We simultaneously obtained an anterior nasal sample for the antigen test and a nasopharyngeal sample for the PCR examination. All samples were obtained with FLOQSwabs (Copan Italia S.p.A., Brescia, Italy).

The anterior nasal sample was initially collected according to the manufacturer’s instructions. Namely, a nasopharyngeal-type flocked (NP-type) swab was inserted to 2 cm depth in one nasal cavity, rotated five times, and held in place for 5 s. The antigen test using the QuickNavi-COVID19 Ag kit was performed immediately after anterior nasal collection and the result was obtained by the visual interpretation of each examiner.

A nasopharyngeal sample was subsequently collected with an NP-type swab according to a previously described procedure^7^ and was diluted in 3 mL of Universal Transport Medium (UTM) (Copan Italia S.p.A., Brescia, Italy). The UTM was transferred to an in-house microbiology laboratory located next to the drive-through sample-collecting site of the PCR center within an hour of sample collection.

### SARS-CoV-2 detection using reverse transcription PCR

After the arrival of the UTM samples, purification and RNA extraction were performed with magLEAD 6gC (Precision System Science Co., Ltd., Chiba, Japan) from 200 µL aliquots of UTM for in-house reverse transcription (RT)-PCR on the same day as sample collection. RNA was eluted in 100 µL and stored at − 80 °C after the in-house RT-PCR test. The eluted samples were transferred to Denka Co., Ltd., every week for a reference real-time RT-PCR test on Applied Biosystems QuantStudio 3 (Thermo Fisher Scientific Inc., Waltham, MA, USA) using a QuantiTect Probe RT-PCR Kit (QIAGEN Inc., Germantown, MD, USA) and primer/probe N and N2 set^8^.

The presence or absence of SARS-CoV-2 was defined by the results of the reference real-time RT-PCR test. However, if discordance existed between the reference real-time RT-PCR test and the in-house RT-PCR test, a re-evaluation was performed with an Xpert Xpress SARS-CoV-2 and GeneXpert System (Cepheid, Sunnyvale, CA, USA), the results of which provided the final judgment.

### The degrees of coughs or sneezes and the severity of pain induced by the sample collection procedure

The degrees of coughs or sneezes and the severity of pain caused by the insertion of the swab into the anterior nasal cavity and nasopharynx in the same participant were assessed. Examiners rated the degrees of coughs or sneezes induction from the following four categories: “None”, “Small, 1–2 times”, “Loud, 1–2 times” and “Loud, multiple times”. The severity of pain was evaluated with a five-point scale (Pain score), with 1 being "no pain" and 5 being “worst imaginable pain,” and the participants were asked to report a number from the scale.

### The comparison of SARS-CoV-2 viral loads between different sample collection sites and swab types

We conducted an additional experiment to evaluate whether the viral loads differed between sample collection sites and swab types between January 8 and 19, 2021. After receiving the participants’ informed consent, two anterior nasal samples were obtained from the participants for whom a nasopharyngeal sample had already tested positive for SARS-CoV-2. Two anterior nasal swab samples were collected from each nostril using one with a NP-type swab and the other with an oropharyngeal-type flocked (OP-type) swab. These sample collections were performed on the same day.

The samples were diluted in 3 mL of UTM, and stored at − 80 °C. After several days of storage, the samples were thawed, and purification and RNA extraction were performed according to the above-described method. The viral concentrations in samples were quantified with the following procedure. The calibration curves were generated with 5, 50, and 500 copies/reaction of positive control (EDX SARS-CoV-2 Standard; Bio-Rad Laboratories, Inc., Hercules, CA, USA). Quantitative RT-PCR was performed on a LightCycler 96 System (Roche, Basel, Switzerland) using a THUNDERBIRD Probe One-step qRT-PCR Kit (TOYOBO Co. Ltd., Osaka, Japan) with a primer/probe N2 set.

### Statistical analyses

The sensitivity, specificity, positive predictive value (PPV), and negative predictive value (NPV) of QuickNavi-COVID19 Ag and their 95% confidence intervals (CIs) were calculated with the Clopper and Pearson method. Categorical variables were assessed by Fisher’s exact test. The viral loads according to collection sites and swab types were compared by the Wilcoxon signed-rank test with Holm correction. The degrees of coughs or sneezes induction and the pain score were also compared between the two different collection procedures using the McNemar–Bowker test and the Wilcoxon signed-rank test, respectively. *p* values of < 0.05 were considered to indicate statistical significance. All statistical analyses were conducted using the R 3.5.2 software program (The R Foundation, Vienna, Austria).

### Ethics statement

The present study was approved by the ethics committee of Tsukuba Medical Center Hospital (approval number: 2020-033). Informed consent was obtained from all participants. We conducted this study in accordance with the Declaration of Helsinki and followed ethical guidelines endorsed by the Ministry of Health, Labour and Welfare, Japan.

## Results

### Viral loads in different sample collection sites and swab types

**Figure 1:**
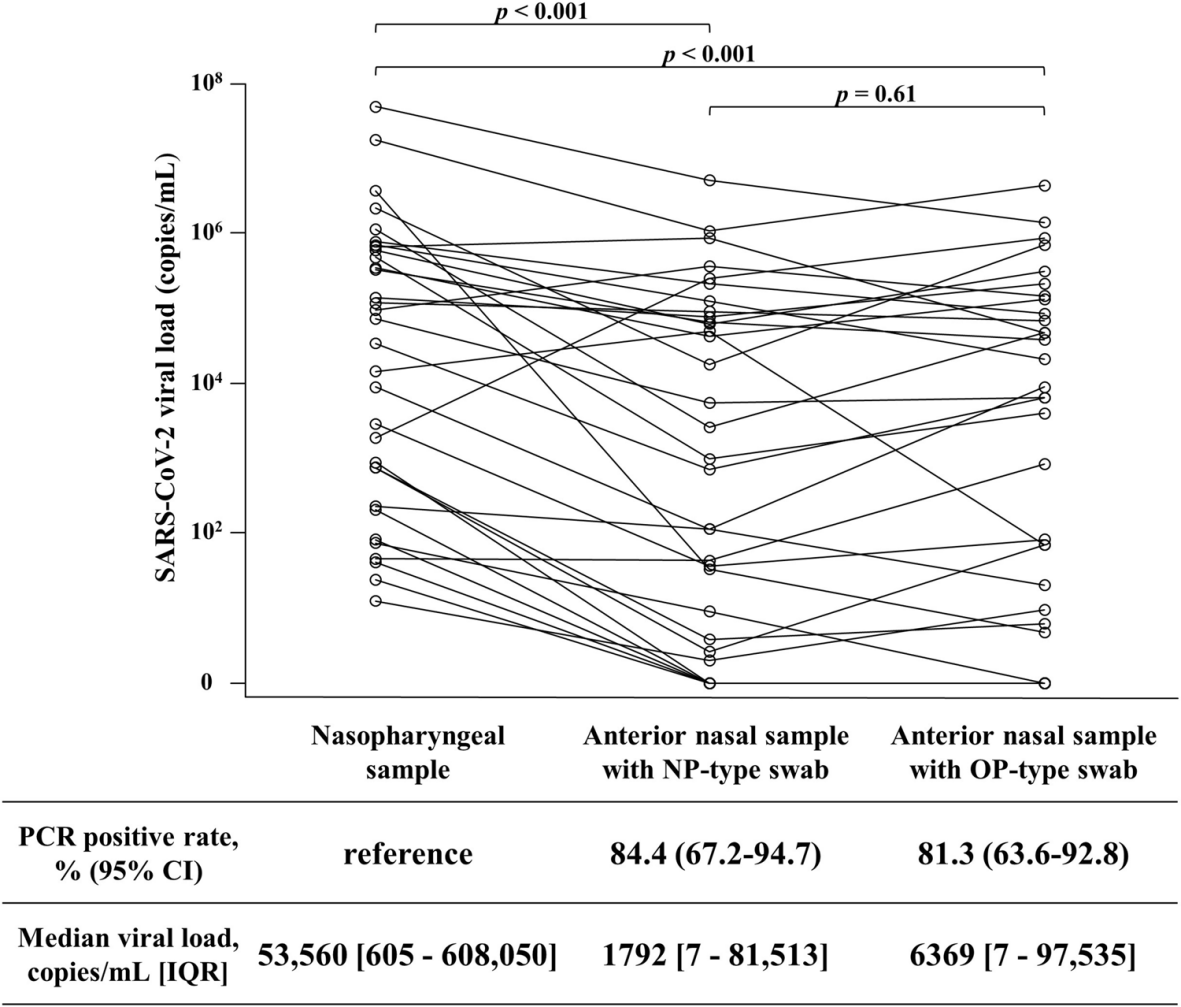
Comparison of SARS-CoV-2 viral loads between each collection site and swab type. The set of viral loads in the same participant is represented by connected lines. The viral loads were compared by the Wilcoxon signed-rank test, and *p* values are corrected with Holm correction. *NP-type swab* nasopharyngeal-type swab, *OP-type swab* oropharyngeal-type swab, *CI* confidence interval, *IQR* interquartile range.

In 32 identical SARS-CoV-2 positive cases, we evaluated the SARS-CoV-2 viral loads of nasopharyngeal samples (NPS), anterior nasal samples with NP-type swabs (AWN), and anterior nasal samples with OP-type swabs (AWO) (Fig. 1). The median viral loads for NPS, AWN, and AWO were 53,560 (interquartile range [IQR] 605–608,050), 1792 (IQR 7–81,513), and 6369 (IQR 7–97,535), respectively. With the NPS as a reference, the PCR-positive rate for AWN was 84.4% (27/32; 95% CI 67.2–94.7%), while that for AWO was 81.3% (26/32; 95% CI 63.6–92.8%). In comparison to NPS, the viral load was significantly lower with AWN (*p* < 0.001) and AWO (*p* < 0.001), but there was no significant difference between AWN and AWO (*p* = 0.61). The viral loads and Ct values for all samples are described in Table S1a and Table S1b.

### Demographic data of study population

A total of 876 participants were screened for the evaluation. Most samples were obtained at the drive-through PCR center, and only 17 were obtained after hospitalization. We excluded the participants who underwent duplicate tests (n = 7) or for whose clinical information were lacking (n = 7). Finally, 862 participants were included in the analysis.

Among the 862 participants, 790 (91.6%) were symptomatic and SARS-CoV-2 was detected in 50 (5.8%) on nasopharyngeal samples by the reference real-time RT-PCR test. The median duration from the onset of symptoms to sample collection was 2.0 days (IQR 1.0–3.0). There was one discordant sample that was positive on the in-house RT-PCR test and negative on the reference real-time RT-PCR test. The Xpert Xpress SARS-CoV-2 test provided a negative result for E target and a positive result of N2 target (cycle threshold [Ct] value of 39.8); thus, 51 (5.9%) participants were finally considered positive for SARS-CoV-2. The discordant sample was obtained from a participant who had been diagnosed with COVID-19 1 month before the current evaluation and who was referred to the PCR center due to refractory respiratory symptoms.

All 51 participants who were positive for SARS-CoV-2 were symptomatic (Table 1); their characteristics are described in Table 2. The most common symptom was fever (80.4%), followed by cough or sputum production (60.8%), sore throat (37.3%), runny nose or nasal congestion (35.3%), and loss of taste or smell (27.5%).

**Table 1:** Demographic data of the whole study population and cases infected with SARS-CoV-2.

	Total	SARS-CoV-2	
		Positive	Negative
N	862	51	811
**Age (years, median [IQR])**	36.0 [24.0, 48.0]	43.0 [30.0, 57.5]	35.0 [23.0, 47.0]
< 18 years	106 (12.3)	2 (3.9)	104 (12.8)
18–64 years	678 (78.7)	44 (86.3)	634 (78.2)
≥ 65 years	78 (9.0)	5 (9.8)	73 (9.0)
Sex (female)	383 (44.4)	19 (37.3)	364 (44.9)
Asymptomatic participants	72 (8.4)	0 (0.0)	72 (8.9)
Symptomatic participants	790 (91.6)	51 (100)	739 (91.1)

*IQR* interquartile range.

**Table 2:** Characteristics of symptomatic participants and cases infected with SARS-CoV-2.

	Total	SARS-CoV-2	
		Positive	Negative
N	790	51	739
Days from symptom onset to sample collection [IQR]	2.0 [1.0, 3.0]	3.0 [2.0, 5.0]	2.0 [1.0, 3.0]
**Signs and symptoms**			
Fever	628 (79.5)	41 (80.4)	587 (79.4)
Cough/sputum production	255 (32.3)	31 (60.8)	224 (30.3)
Runny nose/nasal congestion	185 (23.4)	18 (35.3)	167 (22.6)
Loss of taste or smell	32 (4.1)	14 (27.5)	18 (2.4)
Dyspnea	7 (0.9)	2 (3.9)	5 (0.7)
Fatigue	105 (13.3)	10 (19.6)	95 (12.9)
Diarrhea	48 (6.1)	1 (2.0)	47 (6.4)
Sore throat	210 (26.6)	19 (37.3)	191 (25.8)
Headache	121 (15.3)	11 (21.6)	110 (14.9)

*IQR* interquartile range.

### Diagnostic performance of QuickNavi-COVID19 Ag using anterior nasal samples

Of the 51 participants who were found to be SARS-CoV-2-positive by the RT-PCR test, 37 participants were found to be positive with the antigen test with anterior nasal samples (Table 3). Among the 811 SARS-CoV-2-negative participants, all participants were found to be negative with the antigen test (Table 3). The concordance rate between the antigen test and RT-PCR was thus 98.4% (95% CI 97.3–99.1%). The sensitivity, specificity, PPV, and NPV were 72.5% (95% CI 58.3–84.1%), 100% (95% CI 99.3–100%), 100% (95% CI 86.2–100%), and 98.3% (95% CI 97.2–99.1%), respectively (Table 3). Among the 51 SARS-CoV-2 positive participants, the sensitivities of the antigen test in those with and without runny nose or nasal congestion were 88.9% (16/18, 95% CI 65.3–98.6%) and 63.6% (21/33, 95% CI 45.1–79.6%), respectively. This difference in sensitivity between the two groups did not reach statistical significance (*p* = 0.097).

**Table 3:** Clinical performance of antigen test using anterior nasal samples.

		PCR (nasopharyngeal samples)	
		Positive	Negative
**Antigen test**	Positive	37	0
	Negative	14	811
Sensitivity		72.5 (58.3–84.1)	
Specificity		100 (99.3–100)	
Positive predictive value		100 (86.2–100)	
Negative predictive value		98.3 (97.2–99.1)	

Sensitivity, specificity, positive predictive value, and negative predictive value are provided with 95% confidence intervals.

### Comparison of degrees of coughs or sneezes induction and the severity of pain between anterior nasal and nasopharyngeal sample collection

**Figure 2:**
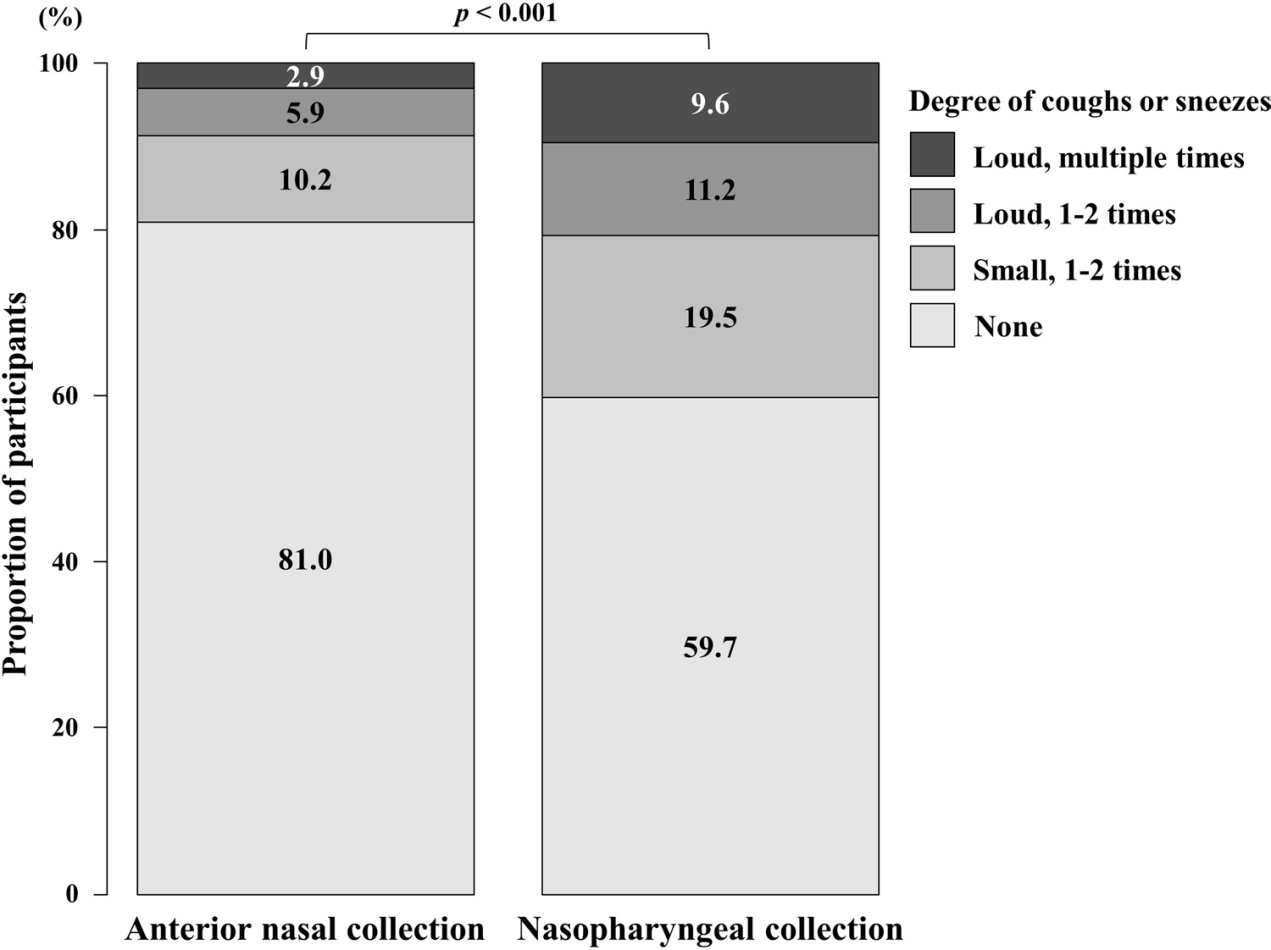
Degrees of coughs or sneezes induced by anterior nasal and nasopharyngeal collection. The degrees of coughing or sneezing following swab insertion was compared between anterior nasal collection and nasopharyngeal collection in the same participant (n = 784). The degrees were rated on a four-point scale. The McNemar–Bowker test was used for the comparison.

The degrees of coughs or sneezes induced by sample collection was measured in 784 participants (Fig. 2). Coughing or sneezing was observed in 149 (19.0%) of anterior nasal collections and in 316 (40.3%) of nasopharyngeal collection. When coughs or sneezes occurred in anterior nasal collection, their degrees were significantly lower than in nasopharyngeal collection (*p* < 0.001).

**Figure 3:**
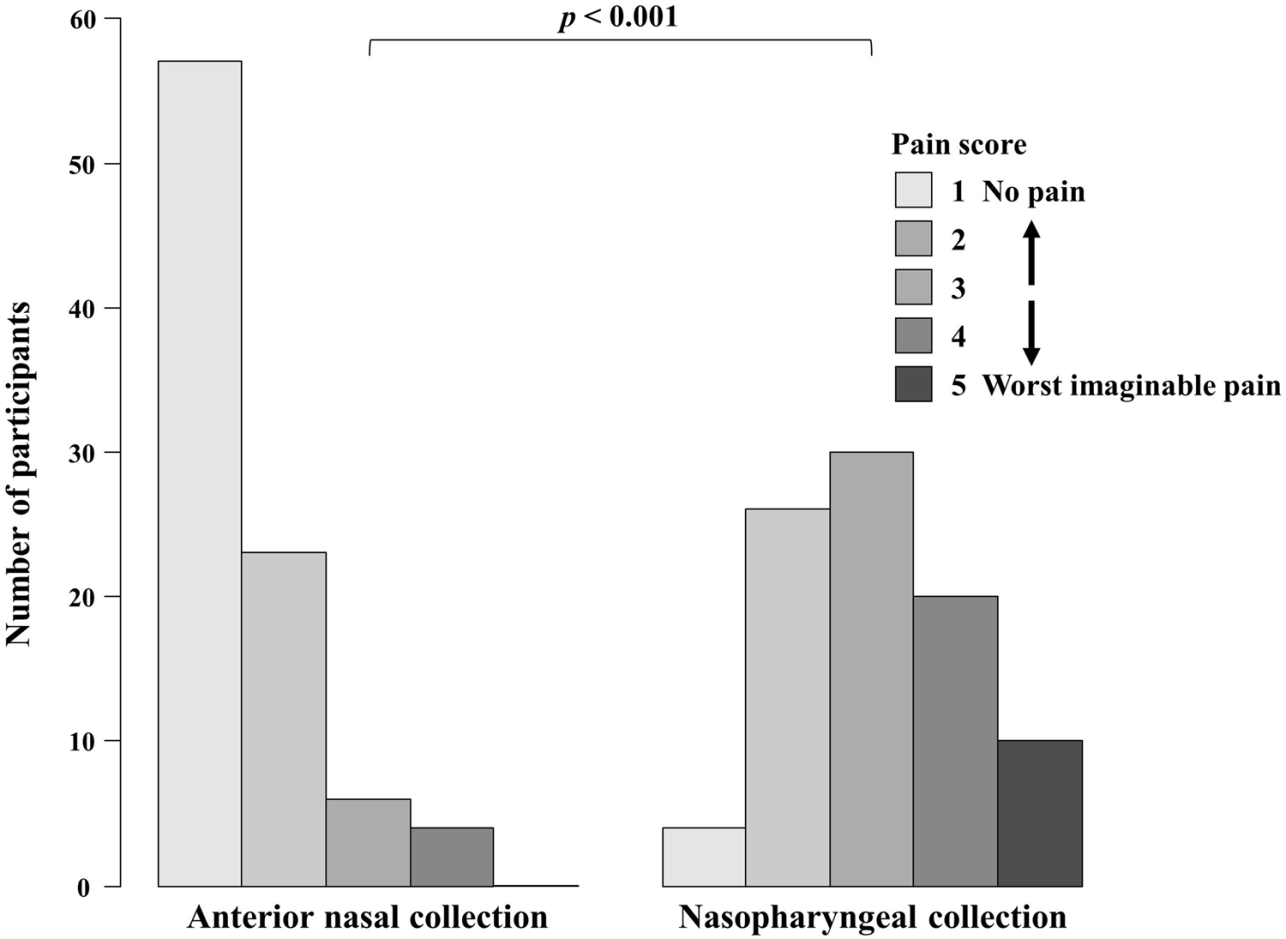
The pain score in anterior nasal collection and nasopharyngeal collection. The severity of pain at swab insertion was assessed on a five-point scale, from 1 to 5 (Pain score). The pain score for each collection method was obtained from the same participant (n = 90). The comparison of the pain scores with the two collection procedures was performed with the Wilcoxon signed-rank test.

The pain score was obtained from 90 participants (Fig. 3). Fifty-seven participants (63.3%) reported no pain in anterior nasal collection. The median pain score of anterior nasal collection and nasopharyngeal collection was 1 (IQR 1–2) and 3 (IQR 2–4), respectively. In comparison to nasopharyngeal collection, anterior nasal collection was significantly less painful (*p* < 0.001).

## Discussion

The QuickNavi-COVID19 Ag test using anterior nasal samples showed 72.5% sensitivity and 100% specificity. In comparison to nasopharyngeal collection, anterior nasal collection was less painful and was associated with fewer coughs or sneezes. In addition, the study demonstrated that the viral load of anterior nasal samples was significantly lower than that of nasopharyngeal samples. Meanwhile, the swab types did not influence the viral load of anterior nasal samples.

Although paired comparison between different swab samples lacked in this study, our study demonstrated that anterior nasal samples provided a lower antigen test sensitivity than our previous study evaluating nasopharyngeal samples^3^. Nevertheless, this lower sensitivity of 72.5% may be acceptable since according to the reported systematic review, sensitivity of nasal swab was 86% in comparison to nasopharyngeal swab by RT-PCR^9^. The sensitivity of antigen tests is largely influenced by the viral load in collected samples^10–13^. The QuickNavi-COVID19 Ag test could detect SARS-CoV-2 in almost all samples with Ct values < 30, and in 18.8% of samples with Ct values > 30^3^. The viral load may vary between collection sites^14^, and this study recognized the viral load of samples was significantly lower when they were collected from the anterior nasal cavity (Fig. 1).

On the other hand, the QuickNavi-COVID19 Ag test provided 100% specificity in both the present study and our previous study^3^. Although it is necessary to verify whether similar results can be obtained in other settings, false positives should be avoided to prevent unnecessary additional testing and inappropriate isolation measures^15^.

We observed that anterior nasal collection caused fewer coughs or sneezes in comparison to nasopharyngeal sample collection (Fig. 2). Notably, anterior nasal collection induced coughs or sneezes in only 19% of participants. Coughs or sneezes generate droplets and prolong their dispersal by forming multiphase turbulent gas clouds^16^, which leads to greater droplet exposure. SARS-CoV-2 is mainly transmitted through droplets^17^; thus, anterior nasal collection, which was associated with fewer coughs or sneezes induction, may reduce the transmission risk among healthcare providers.

Anterior nasal collection was less painful (Fig. 3), with more than half of the participants reporting no pain from the procedure. Nasopharyngeal collection is an uncomfortable and painful experience^6^ and may discourage patients from receiving tests. Besides, nasopharyngeal collection may not be applicable if patients have a history of recent nasal trauma or surgery, remarkable nasal septum deviation, or marked coagulopathy^7^. Despite the decreased sensitivity, when NAATs are not readily available, an antigen test with anterior nasal samples may be an option in these clinical contexts.

The selection of swab type influences the uptake, extraction and recovery efficiency of the collected sample^18,19^. In this study, we compared two flocked swabs with different tip sizes (NP-type and OP-type swab). There was no significant difference in the viral load of the samples collected with the two types of swabs; however, the OP-type swab has a larger tip and seemed to handle a larger amount of samples collected. A previous study suggested that the efficiency of sample release was not associated with the absorbed volume^20^, which could explain the result in this study.

The present study was associated with some limitations. First, the samples used for the reference real-time RT-PCR test were frozen and transported. Although the samples were frozen at − 80 °C, the viral loads may have been decreased during the storage and transport process. Nevertheless, in the case of discrepancy with in-house PCR, re-evaluation was performed and did not affect the calculation of the sensitivity of the antigen test. Second, asymptomatic SARS-CoV-2 positive patients were unintentionally not included in this study. Further study is required to evaluate the clinical performance of the antigen test in those patients. Third, we did not analyze gene mutations of detected SARS-CoV-2. However, according to the manufacturer’s information for use (version 4.0), QuickNavi-COVID19 Ag can similarly react with both variant 20I/501Y.V1, so called a UK variant (VOC 202012/01) and with variant 20J501Y.V3, so called a Brazilian variant P.1 (VOC 202101/02). Finally, this study was conducted at a single center and evaluated a single commercial rapid antigen product. Further research should be conducted to assess the generalizability of the findings.

In conclusion, the QuickNavi-COVID19 Ag test with an anterior nasal sample showed 100% specificity and moderate sensitivity in symptomatic individuals who were in the early course of the disease. Overall sensitivity was lower than the ones observed in our previous study that used nasopharyngeal samples. Anterior nasal collection was less invasive and induced fewer coughs or sneezes, which may be more comfortable for the patient and may reduce the risk of droplet exposure to healthcare workers.

## Supplementary information

Supplementary Informations.

## Data Availability

The data includes sensitive data about the health of human research subjects and thus cannot be directly deposited openly. However, anonymized, individual-level data that enable full replication of the study results are available from the corresponding author.
