# Supplementary material for: Diagnostic performance and characteristics of anterior nasal collection for the SARS-CoV-2 antigen test: a prospective study
Source: Sci Rep. 2021 May 18;11:10519. doi: 10.1038/s41598-021-90026-8 (PMC8131686; doi:10.1038/s41598-021-90026-8)
Supplement: Supplementary file 1 — Supplementary Informations. [file 41598_2021_90026_MOESM1_ESM.pdf]

Diagnostic Performance and Characteristics of Anterior Nasal Collection for the SARS-CoV-2 Antigen Test: A Prospective Study in Japan

Yuto Takeuchi[1,2], Yusaku Akashi[2,3,*], Daisuke Kato[4], Miwa Kuwahara[4], Shino Muramatsu[4], Atsuo Ueda[5], Shigeyuki Notake[5], Koji Nakamura[5], Hiroichi Ishikawa[6], and Hiromichi Suzuki[1,2,7]

[1]Department of Infectious Diseases, University of Tsukuba Hospital, Tsukuba, Ibaraki, Japan

[2]Division of Infectious Diseases, Department of Medicine, Tsukuba Medical Center Hospital, Tsukuba, Ibaraki, Japan

[3]Akashi Internal Medicine Clinic, Kashiwara, Osaka, Japan

[4]Denka Co., Ltd. Gosen site, Reaserch & Development Division, Reagent R&D Depertment, Gosen, Niigata, Japan

[5]Department of Clinical Laboratory, Tsukuba Medical Center Hospital, Tsukuba, Ibaraki, Japan

[6]Department of Respiratory Medicine, Tsukuba Medical Center Hospital, Tsukuba, Ibaraki, Japan

[7]Department of Infectious Diseases, Faculty of Medicine, University of Tsukuba, Tsukuba, Ibaraki, Japan

*yusaku-akashi@umin.ac.jp

Table S1a. SARS-CoV-2 viral load for each sampling site and swab type

Case number	Viral load (copies/mL)		
	Nasopharyngeal sample	Anterior nasal sample with NP-type swab	Anterior nasal sample with OP-type swab
1	755	3	5
2	94,930	357,700	145,400
3	1,804	255,800	851,300
4	730	2	70
5	490,700	962	4,018
6	45	42	818
7	2,851	35	82
8	340,000	41,570	133,100
9	73,090	5,532	6,415
10	14,180	49,480	70
11	2,139,000	18,120	683,000
12	706,800	126,100	21,140
13	588,900	63,240	310,800
14	136,700	78,300	216,900
15	34,030	688	6,322
16	8,616	114	8,763
17	207	Not detected	Not detected
18	119,400	91,150	69,960
19	228	109	19
20	791,900	213,300	85,680
21	886	Not detected	Not detected
22	71	8	Not detected
23	3,773,000	31	4
24	665,500	855,800	48,210
25	327,400	66,120	37,970
26	22	Not detected	Not detected
27	40	Not detected	Not detected
28	79	Not detected	Not detected
29	48,670,000	4,994,000	1,386,000
30	11	1	8
31	17,480,000	1,047,000	4,341,000
32	1,119,000	2,621	48,130

NP-type, nasopharyngeal-type; OP-type, oropharyngeal-type.

Table S2b. SARS-CoV-2 cycle threshold (Ct) value for each sampling site and swab type

Case number	Ct value		
	Nasopharyngeal sample	Anterior nasal sample with NP-type swab	Anterior nasal sample with OP-type swab
1	27.9	35.8	34.8
2	16.6	17.9	19.2
3	24.8	18.4	16.5
4	27.3	35.9	30.8
5	18.3	27.2	25.3
6	31.6	31.5	27.4
7	25.6	31.9	30.7
8	18.9	21.8	20.2
9	21.0	24.7	24.5
10	21.9	19.7	31.2
11	13.1	21.5	15.1
12	15.1	18.1	21.2
13	15.4	19.3	16.5
14	18.0	19.0	17.1
15	20.4	27.4	23.4
16	22.9	30.6	22.8
17	29.5	Not detected	Not detected
18	18.2	18.7	19.2
19	29.3	30.6	33.7
20	14.8	17.2	18.8
21	26.9	Not detected	Not detected
22	34.4	34.3	Not detected
23	14.0	32.1	35.5
24	16.7	16.3	20.8
25	17.8	20.3	21.1
26	32.3	Not detected	Not detected
27	31.5	Not detected	Not detected
28	30.6	Not detected	Not detected
29	12.5	15.6	17.3
30	33.2	36.5	33.6
31	13.9	17.7	15.8
32	17.6	25.9	21.9

NP-type, nasopharyngeal-type; OP-type, oropharyngeal-type.
